# Mitochondrial Mutations and Genetic Factors Determining NAFLD Risk

**DOI:** 10.3390/ijms22094459

**Published:** 2021-04-24

**Authors:** Siarhei A. Dabravolski, Evgeny E. Bezsonov, Mirza S. Baig, Tatyana V. Popkova, Ludmila V. Nedosugova, Antonina V. Starodubova, Alexander N. Orekhov

**Affiliations:** 1 Department of Clinical Diagnostics, Vitebsk State Academy of Veterinary Medicine [UO VGAVM], 7/11 Dovatora Str., 210026 Vitebsk, Belarus; 2 Laboratory of Cellular and Molecular Pathology of Cardiovascular System, Institute of Human Morphology, 3 Tsyurupa Street, 117418 Moscow, Russia; evgeny.bezsonov@gmail.com (E.E.B.); a.h.opexob@gmail.com (A.N.O.); 3 Laboratory of Angiopathology, The Institute of General Pathology and Pathophysiology, 8 Baltiyskaya Street, 125315 Moscow, Russia; 4 Department of Biosciences and Biomedical Engineering (BSBE), Indian Institute of Technology Indore (IITI), Simrol 453552, India; msb.iit@iiti.ac.in; 5 V.A. Nasonova Institute of Rheumatology, 34A Kashirskoye Shosse, 115522 Moscow, Russia; popkovatv@mail.ru; 6 I. M. Sechenov First Moscow State Medical University (Sechenov University), 8/2 Trubenskaya Street, 119991 Moscow, Russia; profmila@mail.ru; 7 Federal Research Centre for Nutrition, Biotechnology and Food Safety, 2/14 Ustinsky Passage, 109240 Moscow, Russia; avs.ion@yandex.ru; 8 Pirogov Russian National Research Medical University, 1 Ostrovitianov Street, 117997 Moscow, Russia

**Keywords:** NAFLD, NASH, chronic inflammation, fibrosis, mitophagy, mitochondrial dysfunction, mitochondrial mutations, oxidative stress, SNPs

## Abstract

NAFLD (non-alcoholic fatty liver disease) is a widespread liver disease that is often linked with other life-threatening ailments (metabolic syndrome, insulin resistance, diabetes, cardiovascular disease, atherosclerosis, obesity, and others) and canprogress to more severe forms, such as NASH (non-alcoholic steatohepatitis), cirrhosis, and HCC (hepatocellular carcinoma). In this review, we summarized and analyzed data about single nucleotide polymorphism sites, identified in genes related to NAFLD development and progression. Additionally, the causative role of mitochondrial mutations and mitophagy malfunctions in NAFLD is discussed. The role of mitochondria-related metabolites of the urea cycle as a new non-invasive NAFLD biomarker is discussed. While mitochondria DNA mutations and SNPs (single nucleotide polymorphisms) canbe used as effective diagnostic markers and target for treatments, age and ethnic specificity should be taken into account.

## 1. Introduction

NAFLD is one of the most common chronic liver diseases worldwide. This disease is multifactorial and complex, involving many aetiological parameters, such as diet, genetic predisposition, and lifestyle, and it is often associated with DM (diabetes milieus), MetS (metabolic syndrome), obesity, and IR (insulin resistance). The main feature is the surplus fat accumulation in the liver (often called hepatosteatosis), not caused by trauma, considerable alcohol consumption, or inflammation. Without medical attention, NAFLD may further progress to NASH, which can be distinguished by chronic inflammation, fibrosis, damaged hepatocytes, and a higher risk of developing HCC and cirrhosis [1]. The NASH stage can develop over months and years; during this period, normal functional tissue is damaged and slowly replaced by scar tissue (fibrosis). Liver performance is decreasing gradually, which eventually often leads to complete failure, at which point liver transplantation may be required [2]. End-stage liver disease such as HCC can develop in NAFLD patients with rather low prevalence (less than 15%); however, such disease’s progression can be independent of cirrhosis status [3].

Today, NAFLD is present in about 24% of the world’s population, among high-risk groups: >80% of obese, 60% of diabetic, and 20% of lean people [4]. However, the worldwide prognosis for NAFLD is negative, suggesting further growth in the number of cases, the increased death rate from cirrhosis, and HCC [5].

Despite its critical importance, the effective treatment for NAFLD is still missing. However, many drugs are ongoing clinical trials [6]. Early and precise diagnostic criteria are crucial for effective therapy, today relying on lifestyle interventions and the amelioration of NAFLD-related complications [7]. The stage of fibrosis is the main marker, used to monitor the progression of the diseases, assign treatment, and predict a long-term outcome [8]. Current methods rely on invasive procedures (like biopsies) and non-invasive methods (blood-markers measurements and image-based). Invasive procedures are expensive and associated with further complications and represent only a small fraction of the liver, which can provide misleading results [9]. Blood-based tests rely on measurements of NAFLD-related markers and general indicators of liver and organism performance. Among others, the most popular criteria are the NAFLD fibrosis score (NFS), AST-to-platelet ratio index (APRI), and enhanced liver fibrosis test (ELF) [10]. Image-based methods (mainly ultrasound, spectroscopy, and magnetic resonance imaging (MRI)) aim to visualize and quantify changes in liver elasticity and hepatic fat content [11]. However, although MRI tools are very indicative, they are expensive and not available for wide application. Modern diagnostic strategies include also “omics” investigation, where lipidomic, proteomic, microRNA, and gut microbiome profiles are used to monitor liver status and disease progression [12].

The current understanding of the NAFLD pathogenesis process relies on the theory of so-called “hits”, where the first “hit” is based on the excess number of FAs (fatty acids) and cholesterol in the liver and circulation. Normally, FAs are metabolized into triglycerides, but due to the impaired β-oxidation, lipotoxic species of saturated FAs and oxidized cholesterol are formed and accumulated in hepatocytes [13]. Subsequent hepatocytes damage leads to apoptosis, necrosis, and necroptosis, causing the release of damage-associated molecular patterns, wide metabolic changes, and formation of the pro-inflammatory environment (known as sterile injury) where activated Kupffer cells and pro-inflammatory (M1-like) macrophages are associated with secreted chemo attractants and stimulating cytokines [14]. However, we should notice that inflammatory response is also an important part of the tissue repair and healing processes; thus, immune cells can release similar mediators in the early stages of liver injury [15]. Thus, immune cells and cytokines can serve as NAFLD/NASH biomarkers; however, obtained information would represent rather system-level inflammation, and every biomarker (or their combination) should be evaluated and supported by other diagnostic tools [16].

In the second “hit”, adipose tissue plays the main role as a source of inflammation [17]. Overrepresented macrophages (such as CCR2+ and CD11c^+^, CD206) can be found in liver and adipose tissue and are accompanied by the release of a high amount of pro-inflammatory cytokines and chemokines in the circulation. This “hit” (often called the adipose tissue–liver axis), together with the progression of liver disease, stimulates the development of IR and powers local and systemic chronic inflammation [18].

In this review, we have analyzed recently identified mutations in mitochondrial DNA and SNPs in nuclear genes, linked with NAFLD. The causative role of mitophagy, mitochondria-delivered ROS, lipotoxic by-products of β-oxidation, and mtDNA in NAFLD development and progression is shown. Additionally, urea cycle metabolites are suggested as a new non-invasive biomarker to evaluate the efficiency of mitochondria performance and the NAFLD stage. The majority of identified SNPs are involved in lipid/glucose metabolism, inflammation, and carcinogenesis. In total, identified SNPs and mtDNA mutations can be used for early NAFLD prediction, diagnosis, and to monitor the progression to more severe forms of the disease (NASH, cirrhosis, and HCC).

## 2. Considering NAFLD/NASH as a Mitochondrial Disease 

### 2.1. Mitochondrial Mutations at the Origins of Liver Inflammation 

Chronic inflammation, fibrosis, and cell death are the main drivers of NAFLD progression [19]. Many studies have supported the view that hepatic steatosis (fat accumulation) can facilitate oxidative stress, inflammation, and activate fibrogenic machinery [20]. Others have focused on the abnormal hepatic FAO (fatty acid oxidation) where mitochondrial malfunctions have been involved [21]. Such mitochondria produce less ATPs and more ROS, and due to the incomplete FAO, also release toxic lipid intermediates that can cause further liver injury [22,23]. Simultaneously, the antioxidant biosynthesis system is not sufficient to neutralize surplus ROS; thus, OS also contributes to the hepatocytes’ stress pathways, inflammation, fibrogenesis, and further NAFLD progression to NASH [24].

Recent research has highlighted that the liver mtDNA of NAFLD patients has a higher mutation rate in comparison to healthy people [25]. The expression analysis of the identified mtDNA mutations and polymorphic sites suggests that the OXPHOS (oxidative phosphorylation) is their primary target, leading to phenotype manifestation. Interestingly, identified mutations were common for the entire body and not somatic, while in patients with advanced fibrosis, damaging somatic mutations have been found in the *mtCYB* gene [25]. The similarity of liver and blood mtDNA mutations suggests that they can be inherited from the mother, not developed de novo. This assumption is supported by many cases of NAFLD development in early childhood when paediatric NAFLD was found to be associated not only with nutrition but also with MetS, T2DM, and the obesity of mothers [26]. In addition to the mtDNA mutations, inherited nuclear mutations may also be the primary cause of OS and higher mtDNA variability. Common POLG missense mutation p.Gln1236His can be a good example, because it is known to deplete the liver mtDNA [25].

Another set of evidence was found in Turkey NASH patients, where the disease progression was associated with nucleotide variations in the D-loop region. It was found that the Mt16318C→A variant was associated with NASH, while the Mt16129AA genotype was associated with more advanced stages of fibrosis. Similarly, other genotypes have been associated with different stages of the disease and other symptoms: the Mt16249 CC—advanced steatosis and lobular inflammation; the Mt16296 TT—hypothyroidism; the Mt16163 GG and Mt16294 TT—MetS; and the Mt16256 TT+CT genotypes—T2DM [27]. 

Accumulation of mutations in the *mtCYB* gene was found to be closely related to the NAFLD severity [28]. The mitochondrial cytochrome b is a fundamental part of Complex III, responsible for the electron transfer from coenzyme Q to cytochrome c, with further proton gradient generation and ATP synthesis. A complex III deficiency can cause different clinical manifestation; depending on the affected tissues, it can be multisystem disorders such as deafness, muscle weakness, cataract, epilepsy, cardiomyopathy, mitochondrial myopathy, and others [29]. NASH patients have a higher *mtCYB* mutations rate, leading to progressive chronic liver degeneration. This process is accompanied by increased release of the oncogenic metabolites (such as 2-hydroxyglutarate), DNA-damaging ROS, lipid peroxyl radicals, and global changes in the liver transcriptome [28].

In summary, mitochondrial mutations play an important role in NAFLD and NASH development and are associated with severe adipose tissue inflammation. Mitochondria are responsible for energy production via β-oxidation of FAs and are also the most important producer of cellular ROS. ROS and other toxic by-products of lipid peroxidation are damaging respiratory chain proteins and mtDNA. Accumulation of mtDNA and surplus ROS production are important hallmarks of NAFLD progression to NASH, cirrhosis, and, finally, liver failure.

### 2.2. Disrupted Mitophagy and NAFLD Progression

Mitophagy is the specialised form of autophagy, targeting the degradation of damaged or malfunctional mitochondria to sustain energy homeostasis. The process of mitochondria turnover via mitophagy is part of many normal physiological events, such as development, cell differentiation, and response to stresses and damage. There are several distinguished stages of mitophagy: initiation, membrane nucleation, phagophore formation and expansion, fusion with the lysosome, and, on the final stage, degradation [30]. In general, mitophagy can be classified as PINK1-dependent and Parkin-in/dependent pathways, while both pathways rely on LC3 (light chain, microtubule-associated protein). LC3 proteins (I and II) are crucial for mitophagy; however, the LC3 deletion can be compensated to restore the normal auto/mitophagy [31]. 

In the PINK1/Parkin pathway, PINK1 senses mitochondrial potential, and, without a specific trigger, is imported to and degraded in the mitochondria. When the mitochondrial potential is out of normal range, PINK1 stabilizes on the OMM (outer mitochondrial membrane) and recruits E3 ubiquitin ligase Parkin. Further, Parkin ubiquitinates several OMM proteins (mitofusins MFN1 and MFN2, VDAC (voltage-dependent anion channel) and others), and initiates phagophore formation and engulfment. Except for Parkin, other E3 ubiquitin ligases can be involved in PINK1-dependent mitophagy (such as SIAH1 and ARIH1). Receptor-mediated mitophagy (Parkin-independent) pathways can be facilitated by OMM (BCL2L13, BNIP3, FKBP8, NIX, and FUNDC1) and IMM (cardiolipin and PHB2) proteins, and some other receptors (p62, OPTN, AMBRA1, NBR1, TAXIBP1, and NDP52) [32].

Several studies have demonstrated a decreased efficiency of mitophagy in the context of aging and an unbalanced diet with NAFLD. Fat-induced liver damage is associated with inhibited mitophagy and following the mitochondria-mediated death of hepatocytes [33]. However, this also suggests several potential mitochondrial targets that can be used as a treatment to reduce inflammation via activated mitophagy, or as a prevention therapy to enhance mitophagy and protect against surplus lipid accumulation in the liver [34,35]. 

Mitochondria use FAO as the main energy source to fuel the cells. Excess substrates for the β-oxidations overload the mitochondrial respiratory chain and favor higher ROS production, which leads to a further dysregulation in mitochondrial functions, structure, transcription, and replication [36]. 

There are several hypotheses explaining the role of mitophagy in the NAFLD/NASH progression. Firstly, hyperlipidemia and hyperinsulinemia are suppressing mitophagy, which leads to the accumulation of lipogenic mitochondria [37]. This hypothesis is supported by the known effect of DM-related drugs, such as liraglutide, which is known to stimulate mitophagy and reduce inflammation and OS production [35]. Thus, effective and timely replacement of lipogenic mitochondria with functional fat-oxidizing mitochondria via the mitophagy process is crucial to prevent NAFLD progression. Additionally, it is known that NAFLD patients have lower acidity of the hepatic lysosomes, which cannot effectively recycle cellular components and sustain normal metabolic functions. As a result, it leads to an increased accumulation of damaged proteins and lipotoxic by-products and gradually increases hepatic TG content [38].

In total, ineffective β-oxidation and increased lipogenesis lead to lipids accumulation in the hepatocytes, ROS generation, and hepatocyte injury, promoting NAFLD progression to hepatic inflammation and fibrosis. This suggests that the stimulation of mitophagy may be a promising therapeutic strategy to limit ROS production and the subsequent death of hepatocytes. 

### 2.3. Non-Invasive Biomarkers to Evaluate the Efficiency of Hepatic Mitochondria

Metabolic abnormalities can be detected in peripheral cells and used as biomarkers to estimate the NAFLD/NASH stage and liver status. The levels of OS and pro-inflammatory cytokines are the main markers to monitor NAFLD severity and progression [39]. Additionally, there are several metabolic pathways completely or partly localized to the mitochondria, such as citric acid and urea cycles. However, under NAFLD conditions, those pathways and their metabolites are compromised and thus can also can provide some insights into NAFLD pathogenesis and new targets for medical intervention. 

**Figure 1:**
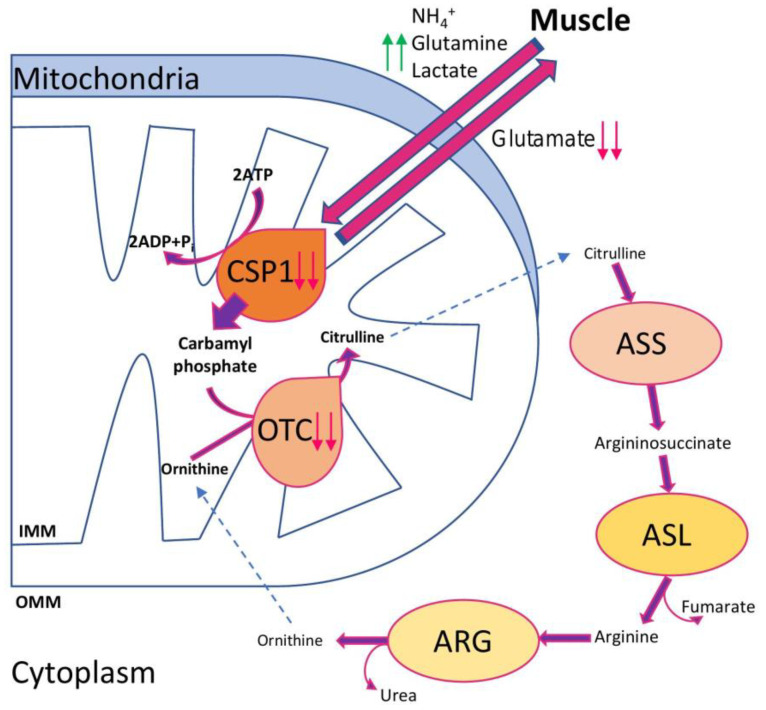
Involvement of the liver mitochondria in ammonia detoxification. Under NAFLD/NASH conditions, the expression and activities of CSP1 and OTC enzymes are reduced (magenta arrows) and the efficiency of the urea cycle is diminished, which leads to hyperammonemia (green arrows) and activation of pro-fibrotic and pro-inflammatory factors favoring the disease progression.

Several mitochondria-related metabolites have been suggested as NAFLD markers: l-ornithine l-aspartate, citrulline/ornithine ratio, glutamine, glutathione, arginine, and glutamate [40]; and CPS-1 (carbamoyl phosphate synthase 1), OTC (Ornithine transcarbamoylase), FGF-21 (Fibroblast growth factor-21), and CK-18 (cytokeratin 18) [40,41]. As it was shown, dysfunctional mitochondria have affected CPS and OTC enzymes, leading to a compromised urea cycle and hyperammonemia (Figure 1). Additionally, hyperammonemia was associated with altered expression of the TLR-pathway, inflammation, increased OS, and hepatocyte apoptosis [42]. The mechanism is likely liver-specific, thus suggesting ammonia as a promising target for NAFLD treatment [43]. FGF-21 is a liver secreted hormone, hepatokine, regulating simple sugar intake in adipocytes and associated with T2DM and NAFLD. FGF-21 has a pleiotropic effect on metabolic homeostasis, which results in increased energy expenditure, fat utilization, and lipid excretion. On the cellular level, FGF-21 enhances mitochondrial oxidative capacity and induces several key metabolic genes via the AMPK/SIRT1 pathway [44]. 

Crucial metabolic pathways, such as the TCA cycle, β-oxidation of FAs, and several steps of the urea cycle are intersecting in mitochondria. The NAFLD progression to more severe forms is most likely based on the direct (from surplus FAs β-oxidation) and indirect (increased OS damage) injury. In this model, accumulated fat and related toxic by-products cause electron transfer chain malfunction, leading to reduced FAD and NAD production, which causes further defects in FAs oxidation and TCA cycle [45]. The particular importance of the urea cycle for the NAFLD progression can be explained by the exclusive location of the rate-limiting steps for ornithine and citrulline metabolism in liver mitochondria. An impaired urea cycle leads to reduced urea synthesis and increases ammonia concentration (hyperammonemia), which is associated with hepatic encephalopathy, inflammation, hepatocyte apoptosis, and high mortality [43]. Additionally, it is known that NASH patients with fibrosis have a much higher level of glutamate, which is also involved in both TCA and urea cycles. Thus, such a connection between TCA and urea cycles suggests that damage to the urea cycle can affect the TCA cycle and provide further cellular damage [46]. 

Those data suggested that pathological changes in mitochondria efficiency can be measured based on the urea cycle metabolites and thus serve as a non-invasive biomarker to monitor NAFLD severity and progression. Further analysis of urea-cycle-related metabolites may provide a better understanding of the involvement of hepatocyte mitochondria in the molecular mechanisms of NAFLD pathogenesis. 

## 3. The Role of OS and Inflammation in NAFLD

NAFLD has become the primary chronic liver disease in both developing and developed countries, mostly due to sedentary lifestyle, lack of exercise, overnutrition, and poor feeding behavior, which are closely related to IR, T2DM, MetS, obesity, and other complications [47]. NASH, a more severe inflammatory form of the disease, in addition to the hepatocyte fat accumulation, requires other aetiological factors, such as mitochondrial oxidative injury. As a by-product of the OXPHOS process, mitochondria generate ROS, which is normally balanced by the scavenging antioxidant system, preventing cell damage [48]. However, an unbalanced ROS-antioxidant system leads to further disruption of mitochondrial functions, impaired mtDNA replication, and transcription, initiating a vicious circle [49]. A high-fat diet and overnutrition are the main triggers of the circle. Thus, excess substrates for the β-oxidations overload the mitochondrial respiratory chain and favors higher ROS production [36]. The current NASH development model suggests that fat-laden hepatocytes have shifted the redox balance with significant ROS overproduction by mitochondria. This model was proven on the experimental animals and humans, where hepatic mitochondrial capacity was challenged with high doses of available lipids [50]. 

### 3.1. OS Activates the NLRP3 Inflammasome and Triggers Chronic Inflammation State

The liver is responsible for the detoxification of harmful substances and endotoxins consumed with diet and created during different metabolic processes. Normally, antioxidants counterbalance ROS generated during detoxification reactions. However, under NAFLD conditions, the antioxidant pool is depleted, while ROS production is greatly increased. As a result, ROS causes damage to DNA, lipids, and proteins, forming oxidative damage products [51]. Following lipotoxic liver injury, ER stress and DNA damage promote further NAFLD inflammation and fibrotic progression [52]. 

#### 3.1.1. The NLRP3 Inflammasome: An OS Sensor and Inflammation Driver

The NLRP3 inflammasome has a sensor (NLRP3), an adaptor (ASC), and an effector (caspase 1). NLRP3 is a three-domain protein, with an N-terminal pyrin domain (PYD), a central NACHT domain, and a C-terminal leucine-rich repeat domain (LRR domain). The self-regulation of NLRP3 relies on the ATPses activity of the NACHT domain, which can be autoinhibited by the LRR domain. An adaptor has two protein–protein interaction domains: N-terminal PYD and a C-terminal CARD (caspase recruitment domain). Caspase 1 has an N-terminal CARD domain and a C-terminal catalytic domain (large and small subunits) [53]. Stimulated NLRP3 can oligomerize via NACHT domain-mediated homotypic interactions with further recruitment of ASC through PYD–PYD interactions. Multiple ASC filaments combine into a speck structure, where caspase 1 is recruited through CARD–CARD interactions and self-activation [54].

Involvement of the NLRP3 inflammasome in an inflammatory process is a two-step process: priming and activation. Priming can be initiated through the PRRs (pattern recognition receptors such as TLRs (Toll-like receptors)), cytokines (TNFα, IL-1β), or NOD2 (nucleotide-binding oligomerization domain-containing protein 2), which activate NF-kB and up-regulate the expression of inflammasome components (caspase 1, NLRP3, and pro-IL-1β). Additionally, priming is stabilizing NLRP3 through PTMs (post-translational modifications): sumoylation, phosphorylation, and ubiquitylation [55].

Inflammasome activation via sensing of cellular stresses is not a fully elucidated process. However, it is known that NLRP3 can be activated by many unrelated stimuli, such as viral, bacterial or fungal infection, but also by internal sterile inflammation and environmental irritants. Among the recognized triggers, we can mention mitochondrial DNA and ROS, ion flux (K^+^, Cl^−^, Ca^2+^), free fatty acids (FAs), and others [56]. While inflammasome description is far beyond the topic of this review, we wish to redirect interested readers to some more focused recent reviews [57,58].

Malfunctional mitochondria are one of the key NLRP3 activators. During cellular stress, ROS production by mitochondria is greatly increased. Mitophagy, as the main remover of damaged and retired mitochondria, is closely connected to the NLRP3 activation. Inhibited mitophagy and stimulated ROS production can significantly enhance NLRP3 activation [59]. FAs are known activators of NLRP3 inflammasome. AMPK, known to suppress inflammation by limiting ROS production and autophagy activation, thus inhibits NLRP3 activation. FAs, however, suppress AMPK and promote NLRP3 activation [60]. 

Activated NLRP3 inflammasome release the pro-inflammatory cytokines IL-1β and IL-18 and initiates pyroptosis, an inflammatory form of lytic programmed cell death. The key player of pyroptosis is gasdermin D (GSDMD), which is activated by caspase 1, binds to the plasma membrane and makes pores, thus, killing cells from within. GSDMD also can bind cardiolipin, presented in bacterial membranes, thus, demonstrating a bactericidal activity. Additionally, GSDMD can facilitate IL-1β and IL-18 release, thus, promoting both chronic and acute inflammatory conditions and leading to the emergence of inflammaging [61]. 

#### 3.1.2. Chronic Inflammation

TLR signalling pathway is one of the key mechanisms involved in low-grade chronic inflammation state in obese, diabetic and NAFLD/NASH individuals. FAs and their derivatives enhance TLR4 activity in both liver and macrophages, and, together with oxLDLs, stimulate the NLRP3 inflammasome activation in atherosclerotic plaques. Further, generated ROS and NF-kB enhance the expression of pro-inflammatory genes. In addition to FAs, many derivatives (such as cholesterol crystals and ceramides) can prime and activate NLRP3 [62]. Prolonged TLR activity is leading to the chronic inflammation state and triggering the development of metabolic syndrome (MetS), which also fuels the inflammation pool, making this vicious cycle complete [63]. 

Endoplasmic reticulum (ER) stress is another pathway, linking inflammation, hepatocyte death and NAFLD progression. Severe ER stress can activate NLRP3 inflammasome and induce the inflammatory process, thus initiating and aggravating chronic diseases [64]. Chronically unresolved ER stress activates the so-called unfolded protein response (UPR), which uses chaperons to regain functional and properly folded proteins. UPR triggers a set of signalling pathways, leading to JNK phosphorylation and apoptosis, but also induction of NF-kD with the production of pro-inflammatory cytokines and following insulin resistance [65]. The UPR’s signalling mechanism is based on IRE1 (inositol-requiring enzyme 1) and transcription factor ATF6 (activating transcription factor 6). The IRE1 activates the NLRP3 inflammasome in the ASC-independent pathway and causes caspase-2-driven mitochondrial damage [66].

ER stress in combination with oxLDL up-regulates CD36 in macrophages, enhancing foam cell formation and thus promoting atherosclerosis progression and plaque necrosis [67]. In mitochondria, CD36 and oxLDL mediate the metabolic switch from oxidative phosphorylation to superoxide production and NF-kB activation. Thus, the CD36/oxLDL signalling pathway links FAs dysregulation, mitochondria ROS production, and chronic inflammation [68]. 

In total, the NLRP3 inflammasome itself, its upstream activators, and its downstream effectors are the main consequences of the OS and can serve as potential new targets against chronic inflammatory diseases such as NAFLD and related co-morbidities (atherosclerosis, MetS, IR, DM, and others).

## 4. Genetic Determinants of the NAFLD Pathogenesis

NAFLD pathogenesis includes environmental, behavioral, and genetic factors. Environmental and behavioral (such as nutrition, physical activities, feeding behavior, and pollutant levels in the environment), genetic (single nucleotide polymorphisms (SNPs) and mutations), and epigenetic (DNA methylation, expressed miRNAs, and histone modifications) factors interact and modulate individual risk of NAFLD development and the severity of progression [69]. It has been known for decades that some NAFLD susceptibility caused by genetic and epigenetic factors can be inherited [70]. Recent GWAS (genome-wide association studies) helped to define particular genes linked with the risk of NAFLD development [71] and NAFLD prevalence in different ethnic groups [72].

Candidate gene studies involve a detailed investigation of a small sample size, where a gene with known function is compared in NAFLD cases and healthy controls. GWAS requires huge sampling but is able to provide genetic association. During the past decades’ many genes, SNPs and epigenetic factors have been linked to NAFLD [69,73]. We wish to redirect interested readers to these reviews; here, we will further discuss recently identified mutations and SNPs (Table 1). 

In general, identified SNPs and genes, associated with NAFLD/NASH, can be assigned to three groups: lipids/glucose metabolism-related, immune/inflammatory response, and other genes. Further, we will discuss several genes with well-studied functions and roles in different diseases.

### 4.1. SNPs in Genes Related to the Lipid/Glucose Metabolism

*PNPLA3* (the Patatin-like phospholipase domain containing 3) gene is involved in the LD (lipid droplets) metabolism and VLDL (very-low-density lipoprotein) secretion; this gene’s SNP site was linked with NAFLD and studied by many researchers around the globe. The role of polymorphism in the *PNPLA3* was shown to be the most crucial factor influencing the ethical differences in hepatic fat content [135]. In particular, the rs738409 polymorphism site (I148M substitution) was studied in several ethnic groups and found to be related to a higher risk of NAFLD development, elevated liver enzymes, and fibrosis [76,82,124,136]. Interestingly, the mitochondrial genome also plays a crucial role in NAFLD/NASH development and progression. As it was shown, the presence of haplogroup L has a protective role against NASH development [137]. There were several molecular mechanisms suggested, explaining the effect of I148M PNPLA3 substitution. The first one implies the involvement of PNPLA3 in the regulation of lipophagy, a specialized form of lipid droplets autophagy. PNPLA3 interacts with LC3-II, the central protein in autophagosome biogenesis, while in M148-PNPLA3 the level of lipophagy was decreased, which leads to slower LDs turnover and higher accumulation of intracellular lipids [138]. Another pathway links inflammation to the NAFLD progression, due to the higher inflammatory infiltration and liver damage found in NAFLD patients with PNPLA3 I148M mutation [139]. It was shown on HepG2 cells culture that NF-kB, the most crucial inflammation-regulating transcription factor, regulates *PNPLA3* expression. Thus, the I148 PNPLA3 protein participates in palmitic-acid-induced inflammatory response through the ER stress pathway and increased TNF-α expression [140].

*PON1* is the well-known member of the paraoxonase (PON) family, associated with HDL and acting like antioxidant-inhibiting LDL oxidation, thus having antiatherogenic effects [141]. The PON enzymes play an important role in lipid and glucose homeostasis and aging and are associated with different metabolic disorders: DM, NAFLD, CVD, neurodegenerative disease, and cancer [142,143]. Recently, *PON1* genetic polymorphism and activity were linked to coronary heart disease in aged patients with confirmed DM; thus, PON1 can be considered as an additional diagnostic factor to evaluate cardiovascular risk [144]. Rs854560 polymorphism (L55M substitution) and low serum PON1 concentration were identified in NAFLD patients, suggesting their potential application in NAFLD early prediction and non-invasive diagnostics [74].

Perilipins (PLINs) proteins are the most abundant lipid droplet proteins, responsible for lipid storage. *PLIN2* gene is constitutively expressed and correlates with lipid droplet density and TG content. Interestingly, plin2^-/-^ mice have much lower TG content and are protected against fatty liver disease [145]. Recently identified among American NASH patients, the rs35568725 polymorphism site (Ser251Pro substitution) was suggested as a risk factor for NASH due to the effect on LD phenotype [75].

Recently, apolipoprotein was suggested as a diagnostic and therapeutic target of NASH. It was noticed that NASH patients have a tight correlation between abnormal apolipoprotein, increased liver fat content, and VLDL plasma concentration [146]. Interestingly, several polymorphism sites (rs10750097, rs1263173, rs17120035, and rs662799) of the *APOA5* gene have been associated with NAFLD in the Chinese Han population [90]. Additionally, the relation of the *APOC3* and *APOE* genes’ polymorphism sites to NAFLD was identified for other ethnic and age groups. The most probable mechanism is based on the role of the apolipoproteins in the accumulation of TG, which leads to lipotoxic liver injury [147].

The *TM6SF2* gene (transmembrane 6 superfamily member 2) regulates VLDL metabolism via the reduction of apolipoproteins secretion. Polymorphism sites influence TM6SF2 protein’s stability and turnover, causing impaired ER-to-Golgi trafficking of VLDL particles and their accumulation in ER. *TM6SF2* variants have been found in several ethnic groups and linked to lipid metabolism diseases [148]. Similarly, a variant of the *FNDC5* gene, generating the soluble protein irisin, has altered protein stability, which is also influenced VLDL metabolism via apolipoprotein B gene regulation. An unstable FNDC5 variant resulted in increased steatosis, insulin resistance, decreased autophagic flux, and hepatocyte death [149].

*PPARγ* (peroxisome proliferator-activated receptor γ) is an important transcription factor, regulating many genes responsible for different aspects of cellular metabolism, differentiation, development, and tumorigenesis [150,151]. Four polymorphism sites (rs9817428, rs1175543, rs13433696, and rs2920502) of the *PPARγ* gene have been identified as NAFLD-related [99]. Since *PPARγ* expression level is increased in NAFLD, obese, and T2DM patients, it was suggested that *PPARγ* acts as a pro-steatosis factor via the de novo lipogenesis and activation of lipogenic genes [152,153].

Adiponectin is a protein hormone secreted from adipose tissue and regulating glucose levels and FAs breakdown. Impaired adiponectin regulation/activity was connected to several metabolic diseases (T2DM, atherosclerosis, NAFLD, obesity, and MetS). Adiponectin can act synergistically with leptin, the other hormone, regulating energy balance and modulating insulin resistance [154]. Thus, it is not surprising that NAFLD-related polymorphism sites also have been found in insulin-related genes, such as *IRS2* (insulin receptor substrate 2) and *IGF1* (insulin-like growth factor 1). In addition to the direct and genetic interaction between glucose and lipid metabolism-regulating genes, *IRS2* also mediates the signalling of IL-4 and interacts with SOCS1 (suppressor of cytokine signaling 1), thus connecting the energy metabolism gene pool with the inflammatory/immune response genes [155].

In summary, defined dysregulation in lipid metabolism provides a complex influence on an organism and, in combination with IR and other factors, significantly increases the risk of a life-threatening co-morbidity such as atherosclerosis [156].

### 4.2. SNPs in Genes Related to the Immune/Inflammatory Response

There were several SNPs sites identified in genes associated with the immune functions and inflammatory response, primarily cytokines: *IL-6*, *IL-6R*, *IL-27*, *IL-17A*, *IL-17RA*, and *TNF-*α. IL-6 is the best-studied pleiotropic pro-inflammatory cytokine, associated with chronic inflammation and the development of many diseases: Alzheimer’s, Crohn’s, anaemia, rheumatoid arthritis, inflammatory bowel disease, cancer, multiple sclerosis, aging, and others [157,158]. The IL-6 receptor is the main interaction partner for IL-6, which activates several signalling pathological pathways (Ras/MAPK, PI3K–PKB/Akt, and JAK/STAT3) and regulates levels of VEGF and CD4+ T cells to execute its biological functions [159]. It is also known that, in the absence of inflammation, up to 30% of IL-6 can be produced by adipose tissue [160], which makes IL-6 the main chronic inflammation factor for diseases linked with the accumulation of surplus fat. In addition to the pro-inflammatory role, IL-6 acts as an anti-inflammatory myokine, produced from muscles upon contraction [161]. As an anti-inflammatory myokine, IL-6 inhibits IL-1 and TNF-α and activates IL-10 and IL-1ra. TNF-α is another cytokine with a defined SNP site (rs1800629), responsible for immune cells’ regulation and implicated in many human diseases and disorders (cancer, psoriasis, cognitive deficits, inflammatory bowel disease, Alzheimer’s, depression, and others) [162,163]. In addition to the role of the pro-inflammatory response mediator, IL-17A and its receptor IL-17RA have many immune regulatory functions, associated with allergic responses and the production of many other cytokines (IL-6, IL-1β, TNF-α, and β), chemokines, and prostaglandins [164]. IL-17 was linked to several immune/autoimmune diseases (lupus, asthma, rheumatoid arthritis, psoriasis, multiple sclerosis, and others) [165].

In total, cytokines have connected into a close co-regulation circuit, creating a self-promoting chronic inflammation micro-environment suitable for the development of many diseases, including obesity, liver diseases, and several types of cancer [166].

### 4.3. SNPs in Other Genes

Among other genes that were found to be associated with NAFLD, we wish to discuss genes associated with tumorigenesis and inflammation. The polymorphism site rs2303861 was identified in the *CD82* (*KAI1*) gene [123], responsible for the downregulation of tumor progression of human cancers, immunity, inflammation, and cognitive function [167,168,169,170]. As it was shown on the mouse model of rheumatoid arthritis (chronic autoinflammatory joint disease), the level of CD82 is increased in RASF (rheumatoid arthritis synovial fibroblasts), where it plays an important role in cell adhesion and motility. RASFs are active cells, migrating and promoting joint inflammation and destruction in non-affected areas [171].

Telomerase reverse transcriptase (TERT) has NAFLD-related substitution C228T [119]. Telomerases are responsible for the regulation of telomeres length, so that senescent cells can potentially become immortal and turn into cancerous cells [172]. In the case of NAFLD, C228T TERT mutations have been suggested as a non-invasive diagnostic biomarker for the disease progression to HCC [173,174]. While the results are very promising, they have been obtained on a small group of patients, so wider studies required before this method can be introduced to the clinical practice.

Sam50, an important mitochondria outer membrane protein, encoded by the *SAMM50* gene and involved in the regulation of mitophagy, mitochondrial morphology and removal of ROS [175]. Several SNPs were linked with NAFLD; however, the exact mechanism, leading to hepatic lipids accumulation and NAFLD progression was not known. *SAMM50*-knockdown cells have lower levels of FAO, ETC activity; however, those effects can be reversed by overexpression of *PPARα*, which is known to enhance FAO [125]. While these results provide direct mechanistic evidence for the involvement of SAMM50 in lipid metabolism, wider research on different ethnic groups required to prove the causative role of *SAMM50* polymorphisms in NAFLD susceptibility.

Interesting results, obtained by Rausch et al., 2018 [101] have suggested several new associations between NAFLD and obesity, DM and IR. However, the most important point of this GWAS study is the absence of *PNPLA3* gene polymorphism, which was associated with NAFLD in several other cross-ethnic studies. This can be explained by a specific cohort (Hispanic boys, up to 18 years of age). However, both aspects of the newly identified SNPs (ethnic and age specificity) required further investigation.

Vitamin D deficiency was linked to NAFLD progression; thus vitamin D supplementation was suggested as an effective NAFLD treatment [176]. Polymorphisms of Vitamin D metabolism-related and signalling genes were associated with diabetes 1 and 2 types and BMI [177,178,179]. Recently, polymorphism site rs1544410 of the Vitamin D receptor was linked to advanced liver fibrosis in Japanese NAFLD patients [130]. Interestingly, the involvement of the liver–gut microbiome axis was suggested as a NAFLD progression factor, acting through nutrient uptake from the diet and bile acid circulation [180].

Another interesting SNP site was identified in NPY (Neuropeptide Y), which is a six-amino-acid peptide neurotransmitter, expressed by chromaffin and noradrenergic cells. It was suggested that high *NPY* expression in the hypothalamus is related to the development of T2DM, IR, and obesity [181]. Probably, the rs16147 variant has altered interaction properties with transcription factor and other regulatory elements. In the case of obese NAFLD patients, this polymorphism site was connected with a lower percentage of steatohepatitis and lobular inflammation [132]. The molecular mechanism of NPY activity relies on the up-regulation of the SREBP2/HMGCR pathway, where SREBP2 is a crucial transcription factor, regulating cholesterol homeostasis, and HMGCR is the rate-limiting enzyme in cholesterol synthesis [182].

In total, we can conclude that many genes have polymorphism sites linked to NAFLD and, potentially, can be used as diagnostic biomarkers for early disease diagnostics, monitoring its progression to NASH and HCC. However, more wide population analysis and studies on different ethnic and age groups are required for successful clinical application.

### 4.4. Functional Association between NAFLD and PNPLA3 Risk Allele

In the last decade, our understanding of the NAFLD pathogenesis has significantly expanded. While tens of SNPs were identified as a NAFLD-development risk factor, only some of them have been studied in detail and assigned to a particular mechanism. Thus, *PNPLA3*, *TM6SF2*, *GCKR*, *MBOAT7*, and *HSD17B13* are the most frequent genes, and their association with NAFLD development was shown for different ethnic groups [183].

However, the primary role in the NAFLD development among non-obese patients was suggested for the *PNPLA3* gene, even further elaborated to the PASH (PNPLA3-associated steatohepatitis) concept [184]. It is necessary to note that the presence of a mutant gene variant is not causing the disease itself. This was shown in Pnpla3^148M^ knock-in mice, which had a normal level of hepatic fat on a standard diet. However, under a high-sucrose diet challenge, the level of hepatic fat was increased two- to three-fold, and PNPLA3 protein quantity on hepatic LDs was 40-fold higher, with no difference in hepatic Pnpla3 mRNA quantity [185]. The exact molecular mechanism of the couriers of the *PNPLA3* risk allele p.148M was further elucidated. Firstly, it was found that the 148M variant can avoid ubiquitylation and proteasomal degradation, which causes accumulation of the mutant protein on LDs [186], where it prevents the TG mobilization by decreasing hydrolysis/transacylation of PUFAs from PUFA-containing diacylglycerols [187]. Interestingly, such detention of the TGs in the liver has a positive effect on the cardiovascular system, because carriers of the 148M *PNPLA3* gene variant have a lower risk of cardiovascular diseases [188].

The mutant variant of the *PNPLA3* gene has a system-level influence on lipid metabolism, causing fat accumulation in hepatocytes and, under certain circumstances, NAFLD development. The *PNPLA3* I148M mutant protein variant is the main common genetic factor, described for several ethnic groups and with well-studied molecular mechanism of action. Such deep and detailed characterization is the first prerequisite for the development of an effective personalized NAFLD treatment. The *PNPLA3* gene variant is widely accepted as the main NAFLD-associated risk factor; however, future research should also consider the role of other genetic risk factors, the majority of which now lack detailed characterization.

## 5. A Bi-Directional NAFLD ↔ DM Relationship

The original connection between liver disease and DM was established in 1889 by Bernhard Naunyn [189] and reviewed in [190]. Since then, many studies have confirmed the close association between NAFLD and DM, MetS, and risk of cardiovascular diseases [191,192,193]. However, the main question of whether NAFLD is a cause or a consequence of DM is still unanswered [194]. Focusing on the different types of DM, we can notice that linkage between NAFLD and DM1T is not so pronounced and depends greatly on the diagnostic strategy (ultrasound, magnetic resonance imaging, biopsy, or transient elastography) with a pooled prevalence of 19.3–22% in adults [195]. Similar results were obtained for the Brazilian population, where NAFLD was diagnosed among DM1T patients with hepatic ultrasound and transient elastography and resulted in 12.6% and 16.8% prevalence, respectively [196]. In contrast, the risk of DM2T and MetS among NAFLD patients was increased two-fold [191]. Similar results were obtained in another study, where the role of abnormal glucose tolerance was examined as a factor, predicting NASH severity in children and adolescents with and without NAFLD. As a result, diabetic and prediabetes conditions were associated with a 2.2-fold increased risk of NASH [197].

Recent research has defined that IR increases circulating ALT (one of the main NAFLD markers). Thus, increased insulin level (due to IR) promotes de novo lipogenesis in the liver and further hepatic IR, with forced stimulation of hepatic gluconeogenesis, leading to the liver’s elevated output of TG and glucose to the circulation. Eventually, those would diminish β-cell function and cause DM2T [198]. In total, IR results in NAFLD, which increases the risk of DM2T, suggesting IR as the main factor connecting NAFLD and DM [199].

The proposed role of IR was supported also by genetic factors, when SNPs in several genes known to define genetic susceptibility to NAFLD were also linked with IR: *PNPLA3*, *TM6SF2*, *GCKR*, *MBOAT7*, and *HSD17B13* (Table 1) [200]. Recently, SNPs in those genes were suggested as the main nutritional sensors, when an individual’s genetic variations impact nutrients utilization and metabolic processes and define the risk of disease development [201]. Among those, *PNPLA3* and *GCKR* SNPs were shown to be the main variants, interacting with insulin, IR, levels of TG, and glucose to increase NAFLD risk in non-diabetic individuals [202]. An even more confident study on the German population suggests that NAFLD cases could be eliminated if the *PNPLA3* mutant variant was absent [203].

The mutant variant of the *MBOAT7* gene, known to localize on the ER–mitochondria membrane border, where biosynthesis of LD and fat occurs, is also regulated by insulin. The strong reduction of *MBOAT7* expression occurs during IR and obesity, leading to altered mitochondrial dynamics and morphology, higher OS, and switches from oxidative phosphorylation toward anaerobic glycolysis [204,205].

In contrast, a recent study on the Japanese NAFLD patients found that the proportions of *PNPLA3* and *KCNQ1* variants did not differ much among the NAFLD non-diabetic and NAFLD diabetic groups. Similarly, the *PNPLA3* genotype does not influence the prevalence of diabetes and incidence of new-onset diabetes [206]. Another study, conducted on Chinese NAFLD patients, found that the *PNPLA3* GG genotype escalates liver steatosis but reduces the risk of DM2T in patients with obesity or IR [207].

In total, we can conclude that NAFLD and DM are closely associated; however, it is not possible to prove causality between them. It was suggested that IR is the main factor linking NAFLD development and increased DM2T risk. Environmental and dietary factors can affect some mutant gene variants and promote NAFLD and DM2T development; nevertheless, some research papers contradict this conclusion at least for Japanese and Chinese NAFLD patients. Further research on different ethnic groups is required to set up a solid connection between NAFLD, increased risk of DM development, and SNPs in related genes.

## 6. Therapy

The molecular mechanisms of NAFLD development and progression are not completely understood, and there is no approved medical treatment for NAFLD [208]. However, current knowledge allows the provision of effective management of NAFLD as a complex metabolic disorder, often associated with IR, obesity, and DM. There are several main targets in NAFLD treatments: (1) lifestyle modification, which includes changes in feeding behavior, healthy diet, and exercise, oriented around weight loss; (2) control and management of the cardiovascular risks (the leading cause of mortality among NAFLD patients); and (3) prevention of the NAFLD progression to more severe forms and development of associated complications [6]. Ideal treatment should combine glucose and lipid-lowering drugs, metabolism modulators, antioxidants, and anti-inflammatory and anti-fibrotic agents [209]. A single substance (natural or synthetic) cannot provide such complex activities; thus, several combined approaches and drugs should be used.

Several recent reviews have covered many aspects of NAFLD treatments, including plant-based compounds [210], DM-targeted drugs, lifestyle modifications, surgeries, and pharmacological approaches [211,212,213]. For those reasons, we will focus on the recent publications discussing NLRP3 inflammasome-oriented treatments (Table 2).

Further, we wish to point to two natural compounds, naringenin and apigenin, with wide therapeutic activities. Naringenin is a flavonoid, widely presented in many fruits and grasses. In addition to its known anti-cancer and antimicrobial properties [222], recent research has defined also liver-specific anti-inflammatory activity [221]. Interestingly, the discovered effect was based on the inhibition of the NLRP3 activation; later, this decreased expression and secretion of IL-1β pro-inflammatory cytokine and lipid deposition in hepatocytes [221]. As a promising NAFLD treatment, naringenin was studied in several model systems and cell lines, proving its high therapeutic potential [223]. Furthermore, it was reported that naringenin has effective anti-diabetic properties, investigated in many in vitro and in vivo studies [224]. Similarly, apigenin is related to the naringenin flavone compound, known historically as a coloring agent. However, now apigenin is known for its wide pharmacological activities on several signalling pathways (NF-κB, JAK/STAT, PI3K/AKT/mTOR, MAPK/ERK, and others); thus, it is an effective suppressor of chronic inflammation-mediated diseases [225] and several types of cancer [226]. Similarly to naringenin, apigenin prevents NLRP3 inflammasome activation and reduces the release of IL-1β and IL-18 cytokines, but also has a direct effect on the mitochondria (discussed earlier ammonia detoxification pathway) manifested via reduced urea acid production [219].

In total, presented data suggest several effective synthetic and natural compounds which can be used for the treatment of NAFLD and related morbidities. Synthetic compounds seem to have more targeted and profound activities; however, their safety (possible cancerogenic and toxic properties, effective doses) requires detailed investigation. Natural compounds, in general, are safe, but, on the other hand, have wider and less specific activities. Further clinical studies are required to define effective therapeutic drugs and their combinations for NAFLD treatment and prevention.

## 7. Conclusions

We can conclude that variability in many nuclear and mitochondrial genes was linked to NAFLD development and progression. This knowledge should be used for the future creation of a comprehensive list of risk factors used for individual NAFLD prediction and, ideally, personalised treatments. NAFLD is triggered by an unbalanced diet, sedentary lifestyle, and absence of physical activity, but it also can be caused by genetic susceptibility because of the inherited mutations. It was shown that hepatic mitochondria have impaired mitophagy and are responsible for exceeding ROS production, the release of lipotoxic by-products of FAs β-oxidation and mtDNA, involved in chronic inflammation, NAFLD progress to NASH, cirrhosis, HCC, and hepatocyte death. Recent results suggest that the efficiency of mitochondria can be examined in a non-invasive way via measurements of urea cycle metabolites and used for NAFLD diagnosis. However, presented data are ethnic-specific and cannot be used as a universal guide. Additionally, some age-dependent factors and co-morbidities can affect NAFLD diagnosis. To overcome this limitation, more cross-ethnic studies with the involvement of various age groups and detailed clinical examination should be conducted in future.

## Figures and Tables

**Table 1:** List of Single-nucleotide polymorphism sites linked to NAFLD/NASH.

Gene	Name	Polymorphism	Substitution	Association with NAFLD	Reference	Patients Notes
Lipids/free fatty acids/glucose metabolism						
*PON1*	Paraoxonase-1	rs854560	L55M	NAFLD development risk factor	[74]	Romanian NAFLD patients
*PLIN2*	Perilipin-2	rs35568725	S251P	NAFLD development risk factor	[75]	American NASH patients
*PNPLA3*	Patatin-like phospholipase domain-containing protein 3	rs738409	444C>GI148M	NAFLD development risk factor	[76]	Turkish NAFLD patients
					[77]	Chinese NAFLD patients
				Associated with NAFLD and NASH susceptibility and progression	[78]	Brazilian NAFLD patients
		rs2281135		Associated with hepatocyte ballooning, lobular and portal inflammation, and NASH	[79]	Indian NAFLD patients
*CDKAL1*	Cdk5 regulatory associated protein 1-like 1	rs10946398	C	Associated with the high triglyceride glucose index and NAFLD development	[80]	Northern Chinese NAFLD patients
*TM6SF2*	Transmembrane 6 Superfamily Member 2	rs58542926	CT/TT E167K	High risk of NAFLD and CRA	[81,82]	Chinese Han NAFLD and CRA patients
					[77]	Chinese NAFLD patients
					[78]	Brazilian NAFLD patients
*HSD17B13*	17β-Hydroxysteroid dehydrogenase type 13	rs6834314	G/G	Increased steatosis but decreased inflammation, ballooning, Mallory–Denk bodies	[83]	Caucasians NAFLD patients
		rs62305723	P260S			
*ABHD5*	Abhydrolase Domain-Containing Protein 5	c.193A>G	T65A	NAFLD development risk factor	[84]	Two Romanian origin sisters, two and five years old, normal BMI
			40G>T; G 14Ter		[85]	Seven families of Italian origin
*CETP*	Cholesteryl ester transfer protein	rs1800777	G/A	Associated with the presence of steatosis and lobulillar inflammation	[86]	Caucasian NAFLD patients
*ADIPOQ*	GBP-28; regulating glucose levels and fatty acid breakdown	rs1501299	G/T	Increased NAFLD susceptibility	[87]	Meta-analysis
		rs11377	C/G	A risk factor for NAFLD development	[88]	Meta-analysis
*LEPR*	Leptin receptor		Q223R	Risk factor for NAFLD in Chinese population	[89]	Meta-analysis
			K109R	Risk factor for NAFLD in Southeast Asian population		
*APOA5*	Apolipoprotein A5	rs10750097	(G/G)	Associated with NAFLD	[90]	Chinese Han NAFLD patients
		rs1263173	(A/A)			
		rs17120035	(T/T)			
		rs662799	(G/G)			
*APOC3*	Apolipoprotein C3	rs2070667	A	Associated with high-grade lobular inflammation in NAFLD patients	[91]	Chinese Han NAFLD patients
			455T>C	associated with NAFLD	[92]	Asian Indian adolescents with overweight/obesity
*APOE*	Apolipoprotein E	rs429358	C	Associated with steatosis and liver damage	[93]	UK biobank samples
*GPAM/* *GPAT1*	Glycerol-3-phosphate acyltransferase	rs2792751	T			
*LAL*	Lysosomal acid lipase	E8SJM-C.894	G>A	LAL activity associated with cryptogenic fibrosis and cirrhosis; not associated with cryptogenic liver steatosis	[94]	Portugal patients with abnormal liver enzymes
*FNDC5*	Irisin, the cleaved extracellular fragment of the Fibronectin type III domain-containing protein 5	rs3480	G	G allele is associated with more severe steatosis in NAFLD Through a microRNA-mediated mechanism controlling FNDC5 mRNA stability	[95]	Caucasian NAFLD patients
			AA	Associated with severe fibrosis in NAFLD patients with sarcopenia	[96]	Chinese NAFLD patients
*GCKR*	Glucokinase regulatory protein	rs1260326	T	y associated with NAFLD among Asian, liver biopsy, adult, and paediatric groups	[97]	Asian NAFLD patients
		rs780094	T	Significantly increased in NAFLD cases		
*NNMT*	Nicotinamide-N-Methyltransferase	rs694539	AA	Significantly correlated with the steatosis degree, NAFLD, and NASH risk factor	[98]	Egyptian NAFLD patients
*PPARγ*	Peroxisome proliferator-activated receptor-γ	rs9817428	C	NAFLD susceptibility	[99]	Chinese NAFLD patients
		rs1175543	G			
		rs13433696	G			
		rs2920502	C			
*ATGR1*	Angiotensin II type 1 receptor	rs1492100	T			
		rs5186	A1166C	A1166C variant affects liver disease, insulin resistance, and endothelial dysfunction in NAFLD	[100]	Non-diabetic Italian NAFLD patients
*SPATS2L*	Spermatogenesis Associated Serine Rich 2 Like	rs295120	A/C	Associated with obesity/adiposity in NAFLD paediatric patients	[101]	Hispanic NAFLD paediatric patients
		rs99521	T/G			
		rs295120	A/C			
*SEMA6A*	Semaphorin 6A	rs2303752	T/C			
*CAMK1D*	Calcium/calmodulin-dependent protein kinase ID	rs17583338	T/C			
*GAS2*	Growth Arrest Specific 2	rs11026723	A/G			
*NCKAP5*	NCK Associated Protein 5	rs12619898	G/A			
		rs17397163	G/A			
		rs11687204	C/T			
		rs17397380	A/C			
Unknown		rs8005339	A/G			
*RFX8*	Regulatory Factor X 8	rs10865041	G/T	Associated with IR in NAFLD paediatric patients		
*FAM19A1*	TAFA Chemokine Like Family Member 1	rs9846667	A/G			
*WBSCR17*	Williams–Beuren Syndrome Chromosomal Region 17 Protein	rs11773571	C/T			
*DZANK1*	Double Zinc Ribbon and Ankyrin Repeat Domains 1	rs4361192	C/T			
*LINC00851*	Long Intergenic Non-Protein Coding RNA 851	rs2295067	A/G			
Unknown		rs8046133	G/A			
*OPCML*	Opioid Binding Protein/cell Adhesion Molecule Like	rs3923850	A/G	DM susceptibility in NAFLD pediatric patients		
		rs11727927	G/A			
Unknown		rs11644684	C/A			
*PEMT*	Phosphatidylethanolamine N-methyltransferase	rs7946	T	Risk of NAFLD development	[102]	Lean-NAFLD Indian patients
*KLB*	Klotho beta	rs7674434	G	Associated with obesity and hepatic inflammation	[103]	Obese/non-obese NAFLD/non-NAFLD Chinese patients
		rs12152703	T			
		rs17618244	G>A	Associated with NAFLD severity in paediatric patients	[104]	Italian NAFLD paediatric patients
*TCF7L2*	Transcription factor 7-like 2	rs7903146	CT + TT	A protective factor against the development of NAFLD	[105]	Chinese NAFLD and CAD patients
			C/T	Associated with NAFLD in Asian Indians	[106]	Non-diabetic Asian Indian NAFLD patients
*SH2B1*	SH2B adapter protein 1	rs7359397	T	Associated with a higher risk of developing a severe stage of NAFLD	[107]	Spanish overweight/obese patients with NAFLD
*IGF1*	Insulin-like growth factor 1	rs6214	AA and AG	Protective effects for NAFLD susceptibility	[108]	Iranian NAFLD patients
*IRS2*	Insulin receptor substrate 2	rs2289046	GG + AG	A marker of decreased NAFLD susceptibility	[109]	Iranian NAFLD patients
*MBOAT7*	Lysophosphatidylinositol acyltransferase 1 (LPIAT1)	rs641738	C>T	Associated with higher liver fat, NAFLD presence, and severity risk factor	[110]	Caucasian adults, a meta-analysis
				NAFLD/NASH development risk factor	[111]	Caucasian NAFLD patients
Immune/inflammatory response						
*IL6R*	Interleukin 6 Receptor	rs2228145	C>A	Associated with NAFLD development in Russian population of Karelia	[112]	Karelian NASH patients
*TNF-α*	Tumor Necrosis Factor α	rs1800629	AG/AA	Associated with NAFLD in the Iranian population	[113]	Iranian NAFLD patients
*IL-6*	Interleukin 6	rs1800795	CG/CC			
			G	NAFLD-associated hyperglycemia in children	[114]	Italian NAFLD paediatric patients
*ANRIL*	P15 Antisense RNA	rs1556516	G			
*IL27*	Interleukin 27	rs4788048		Associated with hepatocyte ballooning, lobular and portal inflammation and NASH	[79]	Indian NAFLD patients
*SOCS1*	Suppressor of cytokine signaling 1	rs243330	1656G>A	Associated with obese NAFLD patients	[115]	Polish NAFLD patients
*PIN1*	Peptidyl-prolyl cis-trans isomerase NIMA-interacting 1	rs2233678	G	Associated with a high NAFLD risk	[116]	Chinese patient case-study
		rs2287839	C			
*IL-17A*	Interleukin-17A	rs2275913	197G/A	Associate with NAFLD development in obese Turkish children	[117]	Obese Turkish children with NAFLD
*IL17RA*	Interleukin 17 Receptor A	rs5748926	T	High NAFLD activity score, a promising biomarker	[118]	eMERGE Network data
Other genes						
*TERT*	Telomerase Reverse Transcriptase		C228T	Catalytic subunit of telomerase; risk factor of NAFLD to HCC promotion	[119]	NAFLD Japanese patients
*TRIB1*	Tribbles-1	rs17321515	A	Associated with the risk of NAFLD in the Chinese Han population	[120,121]	Chinese Han NAFLD patients
		rs2954029	A			
		rs2954021	A	Marker of transition from simple hepatic steatosis into NASH	[122]	American NAFLD patients
*CD82 (KAI1)*	Metastasis suppressor, a membrane glycoprotein	rs2303861	A/G	Associated with the risk of NAFLD in the Iranian population	[123]	Iranian NAFLD patients
*SAMM50*	Sorting and assembly machinery component 50 homolog	rs2143571	G	Associated with the presence and severity of NAFLD in a Korean population	[124]	Korean NAFLD patients
		rs3761472	A			
		rs2073080	T			
		rs738491	TT + CT	Risk and severity of NAFLD in Chinese Han population	[125]	Chinese Han NAFLD patients
		rs2073082	AG + GG			
*PTPRD*	Protein tyrosine phosphatase receptor type D	rs35929428	GA; R995C	Risk factor for NAFLD development, hepatic lipid accumulation, and fibrosis	[126]	Japanese NAFLD patients
*TMPO/LAP2*	Lamina-associated polypeptide-2		InsA;T99fs	Associated with NAFLD; increased lipid droplet accumulation	[127]	Twin-based study
*TLL1*	Tolloid-like 1	rs17047200	AT/TT	advanced risk of fibrosis	[128]	Japanese NAFLD patients
*KCL1*	Kinesin light chain 1	rs4906353	T	Association with a high risk of NAFLD development	[129]	Korean NAFLD patients
*VDR*	Vitamin D receptor	rs1544410	CC	Associated with advanced fibrosis in NAFLD patients	[130]	Japanese NAFLD patients
*MTHFR*	Methylene tetrahydrofolate reductase	rs1801133	C677T	Association with a high risk of NAFLD development	[131]	Chinese NAFLD patients
*NPY*	Neuropeptide Y	rs16147	A	A lower percentage of steatohepatitis and lobular inflammation in obese NAFLD patients	[132]	Spanish NAFLD patients
*ALDH2*	Aldehyde dehydrogenase 2	rs671	GA and AA	Associated with increased probability of NAFLD among Chinese subjects	[133]	Chinese NAFLD patients
*PCSK7*	Proprotein convertase subtilisin/kexin type 7	rs236918	C	Associated with higher triglycerides, aminotransferases, and hepatic inflammation	[134]	Cross-sectional Liver Biopsy Cohort

**Table 2:** NLRP3 inflammasome-targeting NAFLD treatments.

Treatment	Funtion/Target	Line/Mutant	Effect	Reference
Cardiolipin inhibitor shRNA-CLS1	NLRP3 inflammasome	C57BL/6	shRNA-CLS1 treatment significantly reduced the levels of IL-1β and IL-18; NLRP3, ASC, and Caspase-1	[214]
Baicalin, a flavone glycoside	NLRP3 and GSDMD	Human HepG2 cells	Down-regulates NLRP3, Caspase1, ASC, GSDMD, IL-1β, and IL-18	[215]
Benzyl isothiocyanate	NLRP3 inflammasome	C57BL/6	Suppressed lipid accumulation, macrophage infiltration, fibrosis, crown-like structure formation, p20 caspase 1, and p17 IL-1β expression	[216]
Auranofin	NLRP3 Inflammasome	C57BL/6	Decreases the body weight, epididymal fat weight, levels of AST, glucose, triglyceride, cholesterol, and LDL-c; suppressed the expressions of IL-1β, IL-18, caspase-1, NLRP3, NADPH oxidase 4, and PPARγ	[217]
Sweroside	NLRP3 Inflammasome	C57BL/6	Inhibits NLRP3 inflammasome activation by decreasing IL-1β and caspase-1 production; reduces serum AST and ALT levels, hepatic immune cell infiltration, hepatic triglyceride accumulation, and liver fibrosis	[218]
Apigenin	inflammation	C57BL/6	Apigenin reverses activation of the NLRP3 inflammasome, reduces inflammatory cytokines IL-1β and IL-18 released, inhibits xanthine oxidase activity, and reduces uric acid and ROS	[219]
Soluble guanylate cyclase stimulator praliciguat (PRL)	VASP/ NF-κB/NLRP3 inflammasome	C57BL/6	The PRL anti-inflammatory effect was associated with suppression of hepatic levels of IL-1β, NLPR3, ASC, and c-caspase-1. Mechanistically, PRL induces the protein kinase G (PKG)-mediated phosphorylation of the VASP, thus reducing NF-κB activity and Il1b and Nlrp3 gene transcription	[220]
Naringenin; NLRP3 inhibitor MCC950	NLRP3, inflammation	NLRP3−/− HepG2 cells, primary hepatocytes, and Kuffer cells; C57BL/6	Naringenin inhibits activation of the NLRP3/NF-κB pathway, lipid deposition, and IL-1β expression	[221]

## Data Availability

Not applicable.

## Abbreviations

APO	apolipoprotein
APRI	AST-to-platelet ratio index
BMI	body mass index
CK-18	cytokeratin 18
CPS-1	carbamoyl phosphate synthase 1
DM	diabetes milieus
ELF	enhanced liver fibrosis test
FAs	fatty acids
FAO	fatty acids oxidation
FGF-21	fibroblast growth factor-21
FNDC5	fibronectin type III domain-containing protein 5
GWAS	genome-wide association studies
HCC	hepatocellular carcinoma
IMM	inner mitochondrial membrane
IGF1	insulin-like growth factor 1
IL	interleukin
IR	insulin resistance
IRS2	insulin receptor substrate 2
LC3	microtubule-associated proteins, light chain
LD	lipid droplet
MetS	metabolic syndrome
MRI	magnetic resonance imaging
NAFLD	non-alcoholic fatty liver disease
NASH	non-alcoholic steatohepatitis
NFS	NAFLD fibrosis score
NPY	Neuropeptide Y
OMM	outer mitochondrial membrane
OS	oxidative stress
OTC	ornithine transcarbamoylase
OXPHOS	oxidative phosphorylation
PLINs	Perilipins
PNPLA3	Patatin-like Phospholipase Domain Containing 3
PON	paraoxonase
PPARγ	peroxisome proliferator-activated receptor γ
PUFA	polyunsaturated fatty acid
RASFs	rheumatoid arthritis synovial fibroblasts
ROS	reactive oxygen species
SNP	single-nucleotide polymorphism
SOCS1	suppressor of cytokine signaling 1
TG	triglyceride
TERT	telomerase reverse transcriptase
TM6SF2	transmembrane 6 superfamily member 2
TNF	tumor necrosis factor
VDAC	voltage-dependent anion channel
VEGF	vascular endothelial growth factor
VLDL	very-low-density lipoprotein
